# Why do non-urgent patients present to the paediatric emergency department?

**DOI:** 10.1136/bmjpo-2025-003613

**Published:** 2025-08-31

**Authors:** Jennifer Veitch, Jamie Bentley

**Affiliations:** 1 Edinburgh Medical School, The University of Edinburgh, Edinburgh, UK; 2 NHS Lothian, Edinburgh, UK

**Keywords:** Child Health, Health Policy, Qualitative research

## Abstract

Non-urgent presentation to the paediatric emergency department (PED) is an underexplored area within the context of the National Health Service (NHS). Therefore, a pilot study was undertaken in the Royal Hospital for Children and Young People Edinburgh to evaluate the awareness of the unscheduled care policy within NHS Lothian and the reasons why parents/carers bring their child to the PED for non-urgent complaints. It was found that there was a general lack of awareness of the unscheduled care policy, and that non-urgent presentation was often due to inappropriate referral.

Non-urgent presentation to the paediatric emergency department (PED) is an underexplored area within the context of National Health Service (NHS) Scotland. Research elsewhere has shown that non-urgent presentation to the PED contributes to crowding, which can result in long wait times and negative outcomes, such as prolonged pain,^1^ and reduce the quality of care delivered to other patients in the department.^2^

The Scottish Government began a campaign during the COVID-19 pandemic that focused on increasing public health messaging about the existing concept of patients getting ‘the right care in the right place’.^3^ It subsequently became part of NHS Scotland’s recovery plan, which re-outlined the Unscheduled Care Policy and aimed to ‘improve urgent care pathways in the community’.^4^ This was publicised through public health messaging, posters, letter-box distribution and webpage pop-ups.

A pilot study took place at the Royal Hospital for Children and Young People (RHCYP) Edinburgh to evaluate the awareness of the Unscheduled Care Policy within NHS Lothian and the reasons why parents/carers bring their child to the PED for non-urgent complaints.

Between 20 and 28 March 2023, all parents/carers of children (0–16 years) who presented to the RHCYP PED were invited to participate (n=434), except those with children who were: brought by the ambulance service; accompanied by police; critically ill and required immediate care; or in the PED for too short a time to recruit. Those without sufficient English language proficiency to give informed consent were also excluded. Those recruited (n=170) were given access to an online survey made using Jisc Online Surveys. A convenience sample was collected covering in-hours (08:00–18:00) and out-of-hours (18:01–00:00) periods in order to be as representative as possible. Nights (00:01–08:00) were not sampled, as relatively few patients presented during these times.

There was no pre-existing research on this topic within NHS Scotland, so it was important to create a survey that reflected the differences in Scottish policy and answered the study aims. The online survey responses (n=103) were combined with the anonymised patient data relating to their specific presentation from TrakCare (table 1). Each presentation was categorised using International Classification of Diseases 11th Revision (ICD-11). Patients who were admitted, complex, coded under Chapter 22 in ICD-11 (injury/poisoning), under 1 year of age with shortness of breath symptoms, or underwent further investigation/treatment were removed. This created a non-urgent cohort (n=17) that can be defined as: patients who gained no medical benefit from their visit. These patients were reviewed by a PED consultant.

**Table 1:** Characteristics of study population from TrakCare data

	All patients recruited (170)	All survey responders (103)	Non-urgent patients (17)
Age (median)	7.00	9.00	2.75
Female	84	50	7
Male	81	49	10
Missing data	5	4	0
Triage category (median)	4	5	4

Across all parents/carers who completed the survey 9.7% were aware of the Unscheduled Care Policy (n=10), while 55.3% had accessed another service before they attended the PED (n=57). Overall, 50.5% of patients stated being ‘advised to attend by a healthcare professional (HCP)’ as their reason for attendance (n=52), with 34.9% (n=36) accessing their general practice (GP) before their visit. This is compared with the non-urgent group in table 2. It is important that patients (or in this case parents/carers) know the best service to attend not only to minimise wait times, but also so that they can receive the care from the service that is best equipped to deal with their problem.

**Table 2:** Survey results

	All (103)	Non-urgent (17)	P value
Attended another service	57 (53.34%)	13 (76.47%)	0.0505 (>0.05)
**Attended GP before the PED**	**36** (**34.95%**)	**10** (**58.82%**)	**0.03005 (<0.05**)
Advised to attend by HCP	52 (50.48%)	11 (64.71%)	0.13786 (>0.05)
Aware of Unscheduled Care Policy	10 (9.71%)	0 (0.00%)	0.09012 (>0.05)

Bold values are statistically significant.

GP, general practice; HCP, healthcare professional ; PED, paediatric emergency department.

It is also important to understand why other HCPs refer inappropriate patients to the PED. However, due to the lack of integration between documentation systems, it is not easy to answer this question. In Australia, it was found that 30.04% of children presenting to the PED had been referred by their general practitioner, and 25.87% of these referrals (7.77% of all presentations) were judged as not PED appropriate by PED clinicians.^5^ This is similar to the findings from this study. However, it is hard to draw a clear conclusion about why inappropriate referral occurs, particularly as there is no comparable published research relating to Scotland. It could perhaps relate to the fact that over the last decade within the UK, it has been recognised that there is a lack of paediatric-specific training for general practitioners.^6 7^ In addition to this, due to the changing burden of disease within primary care, acute bacterial infections in children are rare, and general practitioners, on average, will only see one child with meningitis every 4–10 years.^7^ This means that during practice placements in primary care, it can be difficult to gain experience of acutely unwell children, and ‘how are general practitioners to learn how to assess for serious illnesses without adequate exposure to unwell children?’.^6^ This could lead to doctors within GP being overly cautious about children with certain types of illness and consequently sending non-urgent patients to the PED, just in case. This lack of familiarity is perhaps reflected in the median age of each cohort as within the non-urgent group it is much lower than both others (table 1).

The application of this research is limited because it was conducted as a single-centre study with a convenience sample. The patient categorisation method used may also make results less reproducible and comparable. In addition to this, the exclusion of injury-related presentations may mean that the study overlooks other potential non-urgent presentations. However, overall, the study shows that there was a lack of awareness about the Unscheduled Care Policy across most participants. Non-urgent presentation was often due to inappropriate referral, which demonstrated that the aims of the policy were not being met. More research is needed to understand if increasing awareness of the Unscheduled Care Policy would alter presentation patterns.
