# Supplementary material for: Why do non-urgent patients present to the paediatric emergency department?
Source: BMJ Paediatr Open. 2025 Aug 31;9(1):e003613. doi: 10.1136/bmjpo-2025-003613 (PMC12406878; doi:10.1136/bmjpo-2025-003613)
Supplement: Supplementary file 1 [file bmjpo-2025-003613.reviewer_comments.pdf]

PEER REVIEW HISTORY

BMJ Paediatrics Open publishes all reviews undertaken for accepted manuscripts. Reviewers are asked to complete a checklist review form and are provided with free text boxes to elaborate on their assessment. These free text comments are reproduced below.

ARTICLE DETAILS

**TITLE (PROVISIONAL)**	Why do Non-Urgent Patients Present to the Paediatric Emergency Department?
**AUTHORS**	Veitch, Jennifer; Bentley, Jamie

VERSION 1 - REVIEW

**REVIEWER NAME**	Gerard Bury
**REVIEWER AFFILIATION**	None disclosed
**REVIEWER CONFLICT OF INTEREST**	N/A
**AI DISCLOSURE**	No
**DATE REVIEW RETURNED**	22-May-2025

**GENERAL COMMENTS**	This appears to be a small scale research exercise, now presented in the form of a letter; as such it should meet similar criteria to similar studies. Issues for clarification:<br><br>1. Full proofing required - several grammatical errors.<br>2. 'Neither patients nor the public were not involved in the design of this study due to time pressure and the scale of the project.' Please explain this sentence.<br>3. It appears that only 17/434 attenders met the authors' criteria for 'non-urgent visits' - surely this low proportion is the main finding of the study?<br>4. It's unclear if the records of the survey responders were accessed - 'The online survey responses (n=103) were combined with anonymised patient data about<br>their presentation from TrakCare (Figure 1)' - or whether a sample of all patient records were accessed. Please clarify and provide evidence of authority and ethical approval to do so.<br>5. Non-urgent and urgent consultations are listed in a table but no statistical comparison is provided - please complete in order to justify conclusions.<br>6. There is a wealth of international evidence on the complexities driving parents/carers to present to an ED. Scottish NHS may have distinct needs and solutions - it's unclear what this exercise adds to the existing literature.

**REVIEWER NAME**	Martina Schmiedhofer
**REVIEWER AFFILIATION**	None disclosed
**REVIEWER CONFLICT OF INTEREST**	N/A
**AI DISCLOSURE**	No
**DATE REVIEW RETURNED**	27-May-2025

**GENERAL COMMENTS**	Thank you for inviting me to review a manuscript covering the important issue of ED user with non-urgent conditions. The introduction, methods and results are clearly presented and well-founded. Due to the limited number of words, the discussion is very short. Nevertheless, it is recommended to make a comment on the general importance of emergency department care within access to the healthcare system and/or to include a comparison with international experiences.<br>Furthermore, an information sheet for participants has been added, which appears to be aimed more at clinicians that patients in the first part.<br>There are a few typos: (p1 l16; p1 line 40) and the readability of the tables would be improved by including percentages.

VERSION 1 – AUTHOR RESPONSE

Reviewer: 1

Gerard Bury

Comments to the Author

This appears to be a small scale research exercise, now presented in the form of a letter; as such it should meet similar criteria to similar studies. Issues for clarification:

1. Full proofing required - several grammatical errors.

- These have been amended.

2. 'Neither patients nor the public were not involved in the design of this study due to time pressure and the scale of the project.' Please explain this sentence.

- I have added an explanation into the piece itself: ‘as it was conducted as a university dissertation project.’

- For additional information, I created and conducted this study as part of my 3rd year intercalated degree and, due to delays receiving ethical approval, had a small window to conduct the surveys because of the deadline set by the University to submit my final written up piece of work.

3. It appears that only 17/434 attenders met the authors' criteria for 'non-urgent visits' - surely this low proportion is the main finding of the study?

- Not all attenders were recruited to the study, as many declined to participate. The patient’s records could only be accessed if their parent/carer had filled out the consent form and had completed the survey, which narrowed the proportion to 17/103 (16.5%). This is comparable to, and in fact higher, studies conducted in other places – such as in an Australian study conducted by Cheek et al which is referred in the piece, that found 7.77% of all presentations were non-urgent. Although this study is small, it was designed to prompt and assist further research in this field rather than to provide a statistically significant large scale study.

4. It's unclear if the records of the survey responders were accessed - 'The online survey responses (n=103) were combined with anonymised patient data about their presentation from TrakCare (Figure 1)' - or whether a sample of all patient records were accessed. Please clarify and provide evidence of authority and ethical approval to do so.

- The records of each patient related to the survey responder was accessed. This was matched with their survey code and anonymised immediately. However, the data remained linked to the survey responses to allow information about the patient to be linked to the reason that they had presented and whether they had accessed another service first. This sentence has been amended to clarify this – 'The online survey responses (n=103) were combined with the anonymised patient data relating to their specific presentation from TrakCare (Figure 1).'

- Local Caldicott Guardian ethical approval was sought and granted as stated in the final paragraph – reference 2336, and I have amended the statement to clarify that this allowed patient information to be accessed.

5. Non-urgent and urgent consultations are listed in a table but no statistical comparison is provided - please complete in order to justify conclusions.

- I have now added p values to Table 2.

6. There is a wealth of international evidence on the complexities driving parents/carers to present to an ED. Scottish NHS may have distinct needs and solutions - it's unclear what this exercise adds to the existing literature.

- I think this piece adds valuable insight into the reasons that this occurs within Scotland, and creates a platform for future research to be undertaken. The international literature has not been undertaken in the same healthcare system as that of the UK and therefore it was important to find out the driving factors within this context.

Reviewer: 2

Martina Schmiedhofer

Comments to the Author

Thank you for inviting me to review a manuscript covering the important issue of ED user with non-urgent conditions. The introduction, methods and results are clearly presented and well-founded. Due to the limited number of words, the discussion is very short. Nevertheless, it is recommended to make a comment on the general importance of emergency department care within access to the healthcare system and/or to include a comparison with international experiences.

- The discussion has been amended to be more expansive and show comparison to an Australian study conducted on the same topic.

Furthermore, an information sheet for participants has been added, which appears to be aimed more at clinicians that patients in the first part.

- The information sheet that was added is identical to that distributed to parents/carers.

There are a few typos: (p1 l16; p1 line 40) and the readability of the tables would be improved by including percentages.

- I have added percentages into Table 2, and fixed the errors that are mentioned.
